# Supplementary material for: Meropenem Stability in Human Plasma at −20 °C: Detailed Assessment of Degradation
Source: Antibiotics (Basel). 2021 Apr 16;10(4):449. doi: 10.3390/antibiotics10040449 (PMC8072937; doi:10.3390/antibiotics10040449)
Supplement: Supplementary file 1 [file antibiotics-10-00449-s001.zip › antibiotics-1164926_Supplementary Materials File S1.pdf]

Detailed bioanalytical method

1.1 Standards and Reagents

Meropenem Trihydrate reference standards and meropenem-D6 internal standard were purchased from TRC (Toronto, Canada). LC-MS grade Methanol, Acetonitrile and Water were purchased from VWR (Leuven, Belgium), Formic Acid from BioSolve Chimie (Valkenswaard, Netherlands). Human plasma were purchased from Red Cross Flanders (Mechelen, Belgium).

1.2 Instrumentation

Shimadzu UHPLC-MS/MS (Ultra-High Performance Liquid Chromatography tandem Mass Spectrometry) consisting of two LC-30AD pumps with a DGU-20A5R degassing unit, SIL-30AC autosampler, CTO-20AC column oven and CBM-20A system controller, coupled with LCMS-8050 triple quadrupole mass spectrometer with electrospray ionization (ESI) probe, was used for the acquisition. LabSolutions software version 6.86 was used for system control and data processes. All concentration calculations were reported using four significant figures.

1.3 UHPLC Conditions

Chromatography separation was carried out on Phenomenex Kinetex™ F5 (100 x 2.1 mm, 1.7 µm) column at 40°C. The autosampler temperature was 4°C. The mobile phase was 0.1% formic acid in water (mobile phase A) and 0.1% formic acid in acetonitrile (mobile phase B), gradient elution (5-55%B) were applied; with a flow rate of 0.4 mL/min and a run time of six minutes. Retention time of Meropenem and its internal standard (IS) was 2.8 min. Typical chromatogram of the analyte and IS of a medium quality control sample is shown in Figure S1.

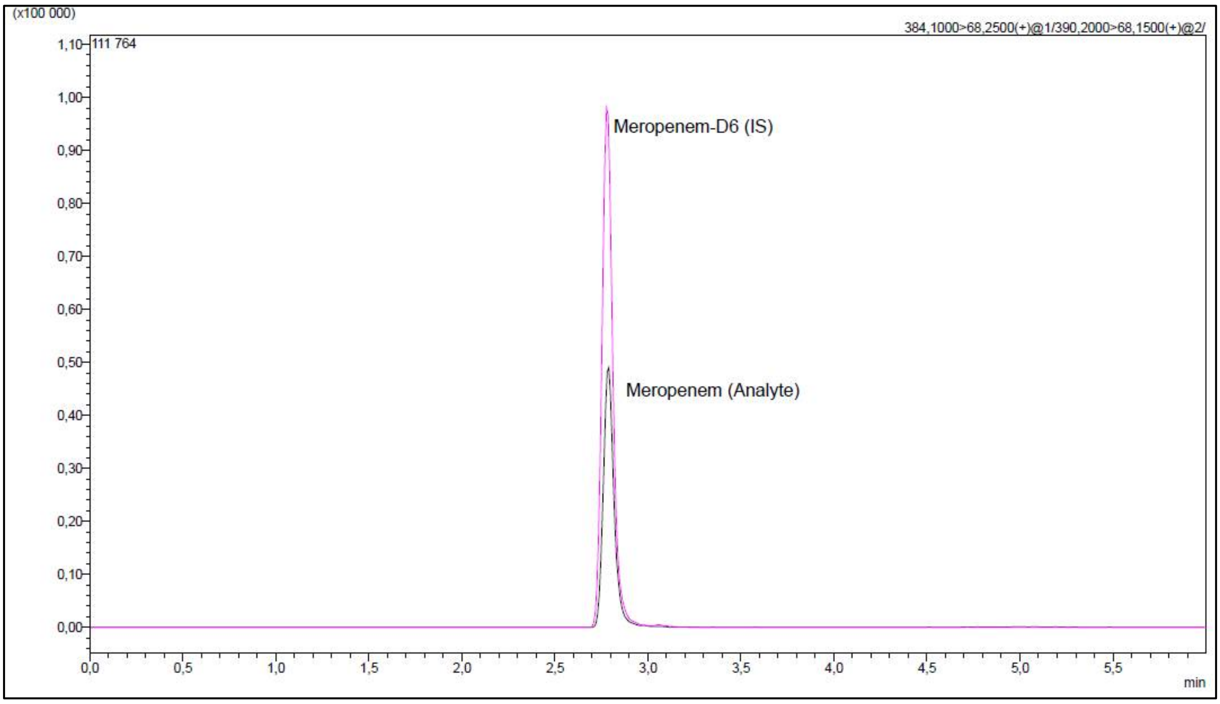

Figure S1. Typical chromatogram of Meropenem and the IS in MQC sample

1.4 Mass spectrometry conditions

The Shimadzu triple quadrupole mass spectrometer was used in positive ionization mode. The ESI interface parameter settings were as follows; interface voltage, 4000V; nebulizing gas flow, 3 L/min; heating gas flow, 10 L/min; interface temperature, 300°C; desolvation Line temperature, 250°C; heat block temperature, 300°C; and drying gas flow, 10 L/min.

Meropenem transitions were 384.1 → 68.25 m/z as quantitative and 384.1 → 141.2 m/z as qualitative transitions with collision energy values of -41.0 and -17.0, respectively; and for the IS (Meropenem-D6) 390.2 → 68.15 m/z as quantitative and 390.2 → 147.3 m/z as qualitative transitions with collision energy values of -39.0 and -14.0, respectively. Dwell time was set to 100 msec for all transitions.

1.5 Preparation of calibration standards and quality control samples

The stock solutions of Meropenem were prepared by dissolving a proper amount of the reference standard in water to obtain a final concentration of 5 mg/mL. Working solutions for calibration curve standards and quality controls (QC) were prepared by diluting the stock solution in water. Final concentrations of Meropenem in human plasma for the calibration standards were 0.1, 0.2, 0.5, 1, 2, 5, 10, 20, 50, and 100 µg/mL. Four QCs, lower limit of quantitation quality control (LLOQ QC), low quality control (LQC), medium quality control (MQC) and high quality control (HQC) levels were used with the concentrations of 0.1, 0.3, 2.5 and 75 µg/mL in plasma. All stock solutions and working solutions were stored

at -20°C. Internal Standard's stock solution was prepared by dissolving a proper amount of Meropenem-D6 in Methanol to obtain a final concentration of 2 mg/mL. Internal Standard was spiked to the plasma samples within the precipitation solution, and the final concentration of IS-precipitation solution was 1.2 µg/mL.

1.6 Sample preparation

Before each analysis, plasma samples were thawed at room temperature. Calibration standards were freshly spiked for every batch. QC samples were prepared by spiking appropriate amounts of working solutions into blank human plasma with 1:19 ratio. Into 100 µL plasma sample, 400 µL of IS-precipitation solution (IS in methanol) was added for protein precipitation and vortex mixed for approximately 2 minutes. Samples were then centrifuged at 11000 rpm for 10 minutes at 4°C, and finally 100 µL of each sample's supernatant was diluted with 1000 µL Water in glass vials and placed in the LC autosampler at 4°C. 1 µL was injected for analysis.

1.7 Calibration curve linearity

Linear regression with $1/x^2$ weighting factor was the most accurate model, which described the concentration-response relationship. The calibration curve was linear over the concentration range of 0.1-100 µg/mL for Meropenem. The calibration curve standard's average accuracy (%) were in the range 92.2 – 104.0%, the highest CV (%) was 5.3% and R^2 were 0.999 - 1.000.

1.8 Precision and accuracy

Three different precision and accuracy batches were analysed on three different days. The CV (%) and accuracy (%) of QCs at each level within each batch (intra-assay) was within acceptable limits for all batches. Mean inter-assay (overall) CV (%) (precision) and accuracy (%) results of 9 QC replicates are presented in

Table .

Table S2. Inter-assay (overall) precision and accuracy results

QC ID	Nominal Conc. (µg/mL)	Mean Calculated conc. (µg/mL)	Accuracy (%)	CV (%)
LLOQ QC	0.1000	0.0961	96.1	8.0
LQC	0.3000	0.2907	96.9	4.7
MQC	2.500	2.446	97.9	3.3
HQC	75.00	73.01	97.3	2.1

1.8.1 Recovery & matrix effect

The recovery of the analyte from human plasma was evaluated by comparing the peak area ratios of four replicates of extracted QC samples, versus the mean peak area ratios of four replicates of post-extraction spiked samples representing 100% recovery. For this experiment the IS was spiked after extraction to compensate for the variation in chromatography and MS/MS detection. The evaluation was assessed at low and high QC concentrations. The recovery of Meropenem was 95.0% - 96.3% for LQC and HQC, respectively, and the CVs (%) of all recovery samples were less than 3.0%.

Matrix effects were also investigated using three lots of blank matrix from individual donors. For each analyte and IS, the matrix factor was calculated for each lot of matrix, by calculating the ratio of the peak area in the presence of matrix (measured by analysing blank matrix spiked with analyte after extraction) to the peak area in the absence of matrix (pure solution of the analyte). The IS normalized matrix factor (MF) was also calculated by dividing the MF of the analyte by the MF of the IS. This determination was done in three replicates at LQC and HQC concentrations. The CVs (%) of IS-normalized MF for Meropenem LQC and HQC samples were 2.6% and 0.8% correspondingly.

1.9 Carry over

The carry over effect was monitored prior to the injection of every analytical run as part of system suitability. Additionally, the carry over effect was investigated by injection of a blank sample after CCS10 sample. CCS10 is the upper limit of quantitation. The maximal carry over for Meropenem was 3.5% in the blank, and the maximal carry over for Meropenem-D6 (IS) was 0.0% in the blank.

1.10 Stability

Since the aim of the study was to assess the stability of Meropenem, the analyte stability was investigated for only sample processing conditions. Results are summarized in Table S. Processed sample stability at room temperature (4 hours) and autosampler stability (4°C nominal for 24 hours) were assessed by three replicates of QC at low and high concentrations. Stability tests were calculated along with freshly prepared and extracted calibration curve and QC samples. In addition, short term stability of analyte in plasma and working solutions at room temperature were investigated during method development, by comparing the peak area ratios of samples left at room temperature for 20 hours, with freshly prepared samples and the difference (%) was -14.3% for analyte in plasma sample, and -9.9% for analyte in working solution. However, during study samples analysis, no sample left for more than 2 hours at room temperature.

Table S3. Stability Results

Stability	QC Level	Nominal Conc. (µg/mL)	Mean Conc. (µg/mL)	CV (%)	Accuracy (%)
Autosampler Stability (24 hours)	LQC	0.30000	0.28090	5.4	93.6
	HQC	75.000	68.817	1.8	91.8
Processed Sample Stability (4 hours)	LQC	0.30000	0.28962	2.8	96.5
	HQC	75.000	72.706	0.5	96.9

1.11 Study samples analysis

Four hundred fifty six study samples were analysed randomly on six different batches, on six subsequent days. Samples were analysed blindly by scanning the QR codes on each sample on the day of analysis, and naming the samples accordingly, then reporting the concentrations of the corresponding QR code. For each analytical batch, freshly spiked

calibration curve standard and four replicates of low, medium and high quality control samples were prepared and analysed. System suitability samples were included and injected at the beginning, in the middle and at the end of every analytical run, to ensure the stability of the instrument. The mean accuracy (%) for the calibration curve during the 6 batches was in the range 96.0 – 103.8% and the CVs (%) of all calibration curve standards were less than 3.9%. R^2 was not less than 0.998. The mean accuracy (%) of QC samples was in the range 97.7 – 101.6% and the highest CV (%) was 3.4%.
