# Supplementary material for: Meropenem Stability in Human Plasma at −20 °C: Detailed Assessment of Degradation
Source: Antibiotics (Basel). 2021 Apr 16;10(4):449. doi: 10.3390/antibiotics10040449 (PMC8072937; doi:10.3390/antibiotics10040449)
Supplement: Supplementary file 1 [file antibiotics-10-00449-s001.zip › Supplementary Materials_Table S1.pdf]

Table S1. Critical threshold values

Table S1. Critical threshold values for each concentration, as simulated based on the polynomial model. The critical threshold corresponds to the duration at which the lower 95% confidence interval of the simulated meropenem degradation at -20°C crosses 85% of the initial meropenem concentration.

Experiment Number	Critical threshold (days)
E1 (0.44 mg/L)	135
E2 (4.38 mg/L)	127
E3 (17.5 mg/L)	105
E4 (35.1 mg/L)	82
E5 (52.6 mg/L)	74
E6 (70.1 mg/L)	76
E7 (87.6 mg/L)	80
